# Psychosocial symptom networks across atrial fibrillation subtypes: evidence from two waves of the ARENA cohort

**DOI:** 10.1093/ehjopen/oeag166

**Published:** 2026-10-08

**Authors:** Stefan Salzmann, Kilian Stenzel, Max Berg, Jochen Senges, Christopher M Celano, Jeff C Huffman, Martin Borggrefe, Ibrahim Akin, Dierk Thomas, Christopher Jan Schwarzbach, Thomas Kleemann, Steffen Schneider, Matthias Hochadel, Tim Süselbeck, Harald Schwacke, Angelika Alonso, Markus Haass, Karl-Heinz Ladwig, Christoph Herrmann-Lingen, Monika Sadlonova

**Affiliations:** Department of Clinical Psychology and Psychotherapy, Marburg University, Gutenbergstraße 18, 35032 Marburg, Germany; Clinical Psychology and Psychotherapy, Vinzenz Pallotti University, Pallottistraße 3, 56179 Vallendar, Germany; Medical Psychology, Health and Medical University Erfurt, Dalbergsweg 5, 99084 Erfurt, Germany; Department of Clinical Psychology and Psychotherapy, Marburg University, Gutenbergstraße 18, 35032 Marburg, Germany; Department of Clinical Psychology and Psychotherapy, Marburg University, Gutenbergstraße 18, 35032 Marburg, Germany; Clinical Psychology Group, Department of Psychology, University of Konstanz, Feuersteinstr. 55, 78479 Konstanz Reichenau, Germany; Stiftung Institut fuer Herzinfarktforschung (Foundation IHF), c/o Klinikum Ludwigshafen, Bremserstraße 79, 67063 Ludwigshafen, Germany; Department of Psychiatry, Massachusetts General Hospital, 55 Fruit Street, Boston, MA 02114, USA; Department of Psychiatry, Harvard Medical School, 25 Shattuck Street, Boston, MA 02115, USA; Department of Psychiatry, Massachusetts General Hospital, 55 Fruit Street, Boston, MA 02114, USA; Department of Psychiatry, Harvard Medical School, 25 Shattuck Street, Boston, MA 02115, USA; Department of Cardiology, Pneumology, Angiology, and Emergency Medicine, University of Mannheim Medical Center, Theodor-Kutzer-Ufer 1–3, 68167 Mannheim, Germany; German Center for Cardiovascular Research (DZHK), Partner Site Heidelberg/Mannheim, Theodor-Kutzer-Ufer 1–3, 68167 Mannheim, Germany; Department of Cardiology, Pneumology, Angiology, and Emergency Medicine, University of Mannheim Medical Center, Theodor-Kutzer-Ufer 1–3, 68167 Mannheim, Germany; German Center for Cardiovascular Research (DZHK), Partner Site Heidelberg/Mannheim, Theodor-Kutzer-Ufer 1–3, 68167 Mannheim, Germany; Department of Internal Medicine III – Cardiology, Angiology and Pneumology, Medical University of Heidelberg, Im Neuenheimer Feld 410, 69120 Heidelberg, Germany; German Center for Cardiovascular Research (DZHK), Partner Site Heidelberg/Mannheim, Im Neuenheimer Feld 410, 69120 Heidelberg, Germany; Department of Neurology, Hospital of Ludwigshafen, Bremserstraße 79, 67063 Ludwigshafen, Germany; Department of Neurology, SRH Gesundheitszentrum Bad Wimpfen, Bei der alten Saline 2, 74206 Bad Wimpfen, Germany; Department of Cardiology, Klinikum Ludwigshafen, Bremserstraße 79, 67063 Ludwigshafen, Germany; Stiftung Institut fuer Herzinfarktforschung (Foundation IHF), c/o Klinikum Ludwigshafen, Bremserstraße 79, 67063 Ludwigshafen, Germany; Stiftung Institut fuer Herzinfarktforschung (Foundation IHF), c/o Klinikum Ludwigshafen, Bremserstraße 79, 67063 Ludwigshafen, Germany; Clinic of Cardiology, Kardiologische Praxisklinik Ludwigshafen, Ludwig-Guttmann-Straße 11, 67071 Ludwigshafen, Germany; Diakonissen-Stiftungs-Hospital Speyer, Paul-Egell-Straße 33, 67346 Speyer, Germany; Department of Neurology, Mannheim Center for Translational Neuroscience, Medical Faculty Mannheim, University of Heidelberg, Theodor-Kutzer-Ufer 1–3, 68167 Mannheim, Germany; Department of Cardiology, Bruederklinikum Julia Lanz, Speyerer Straße 91–93, 68163 Mannheim, Germany; Department of Psychosomatic Medicine and Psychotherapy, University Hospital Rechts der Isar, Technical University Munich, Ismaninger Straße 22, 81675 Munich, Germany; German Centre for Cardiovascular Research (DZHK), Partner Site Munich Heart Alliance, Pettenkoferstraße 8a and 9, 80336 Munich, Germany; Department of Psychosomatic Medicine and Psychotherapy, University Medical Center Göttingen, Von-Siebold-Straße 5, 37075 Göttingen, Germany; German Center for Cardiovascular Research (DZHK), Partner Site Lower Saxony, Robert-Koch-Straße 40, 37075 Göttingen, Germany; Department of Psychiatry, Massachusetts General Hospital, 55 Fruit Street, Boston, MA 02114, USA; Department of Psychosomatic Medicine and Psychotherapy, University Medical Center Göttingen, Von-Siebold-Straße 5, 37075 Göttingen, Germany; German Center for Cardiovascular Research (DZHK), Partner Site Lower Saxony, Robert-Koch-Straße 40, 37075 Göttingen, Germany; Department of Cardiovascular and Thoracic Surgery, University Medical Center Göttingen, Robert-Koch-Straße 40, 37075 Göttingen, Germany

**Keywords:** Network analysis, Atrial fibrillation, Health-related quality of life, Generalized anxiety, Cardiac anxiety

## Abstract

**Aims:**

Atrial fibrillation (AF) carries substantial psychosocial burden, but how psychosocial symptoms interrelate across AF subtypes and severities remains unclear. This study compared psychosocial symptom networks across time, AF type, and European Heart Rhythm Association (EHRA) class in the ARENA (Atrial Fibrillation Rhine-Neckar Region) cohort.

**Methods and results:**

Adults with AF were assessed at baseline (T0) and follow-up (T1). Analytic samples included 996 patients with EHRA class [asymptomatic (EHRA 1) vs. symptomatic (EHRA ≥ 2)] and 924 with AF subtype (paroxysmal, persistent, or permanent) data. Measures included anxiety and worry (GAD-2), cardiac worry and avoidance (CAQ-2), health-related quality of life (HRQoL) (EQ-5D-5L), and psychosocial stressors. Gaussian graphical models were estimated and compared using network comparison test. Global network structures were stable across EHRA classes, AF subtypes, and time points, but specific edges and node centralities varied. The most robust edges linked anxiety with general worry and cardiac worry with avoidance. Anxiety, general worry, cardiac worry, and diminished HRQoL were consistently the most central nodes. Among symptomatic (EHRA ≥ 2) patients, loneliness emerged as an increasingly central node over time, whereas among asymptomatic patients (EHRA 1), sleep problems showed a stronger association with HRQoL at follow-up. Atrial fibrillation–type network structures were similar, although paroxysmal networks showed higher node centrality and stronger anxiety–worry coupling at T0; persistent or permanent AF showed a stress-at-home–cardiac worry edge at T1.

**Conclusion:**

Psychosocial symptom networks in AF are comparable across subtypes and reveal intervention targets, particularly anxiety, cardiac worry with avoidance, loneliness, and sleep disturbance.

Key learning points What is already known

- Atrial fibrillation is associated with impaired health-related quality of life and increased psychosocial burden, with anxiety being one of the most consistently reported psychological correlates.
- Prior observational studies, including analyses from the ARENA cohort, have suggested that generalized anxiety is independently associated with worse health-related quality of life in patients with atrial fibrillation.
- How different psychosocial symptoms interrelate with each other across atrial fibrillation subtypes, symptom severity, and over time has remained insufficiently characterized.

What this study adds

- In a large, prospective atrial fibrillation cohort, psychosocial symptom networks were globally stable across atrial fibrillation subtypes, EHRA symptom classes, and over one year, despite meaningful local differences in specific symptom connections.
- Anxiety, general worry, cardiac-specific worry, and reduced health-related quality of life consistently emerged as the most central symptoms across all network models, suggesting a robust psychosocial core that is largely independent of atrial fibrillation subtype or symptom severity.
- Subgroup- and time-specific shifts in symptom relevance were identified, including increasing centrality of loneliness in more symptomatic patients and stronger links between sleep problems and quality of life in less symptomatic patients, highlighting heterogeneity in psychosocial symptom interrelations within atrial fibrillation.

## Introduction

Atrial fibrillation (AF) is the most common sustained cardiac arrhythmia, affecting millions worldwide and imposing a growing clinical and socioeconomic burden.^1,2^ Beyond its cardiovascular risks, AF markedly impairs health-related quality of life (HRQoL), with symptom frequency and perceived severity contributing substantially to patient distress.^3,4^

Mental health is increasingly recognized as an integral component of cardiovascular health. Recent consensus statements from the European Society of Cardiology emphasize that mental health and cardiovascular disease interact in a multidirectional manner, whereby psychological factors contribute to the development and progression of cardiovascular disease, and cardiovascular conditions themselves adversely affect mental health.^5^ Psychosocial stressors and mental health conditions, including anxiety and chronic stress, have been identified as independent risk factors for cardiovascular morbidity and mortality, with effects comparable to traditional somatic risk factors.^5^

In AF, this interplay may be particularly relevant. Atrial fibrillation is characterized by recurrent and often unpredictable symptoms, which can amplify symptom perception, trigger maladaptive cognitive and behavioural responses such as worry and avoidance, and contribute to reduced HRQoL.^6^ At the same time, psychosocial factors may influence disease perception, coping, and healthcare utilization, thereby shaping the overall disease burden beyond objective arrhythmia characteristics. In this context, treatment guidelines emphasize patient-reported outcomes (PROs) as essential measures of treatment success and highlight the need for integrated approaches that consider biological, psychological, and social determinants of health.^7^ Recent ESC guidelines for the management of AF further emphasize the importance of integrated, patient-centred care, as reflected in the AF-CARE pathway, in which symptom burden and quality of life are central treatment targets.^8^ Building on this perspective, psychosocial stressors—such as anxiety,^9^ loneliness,^10^ or sleep disturbances^11^—are not peripheral consequences but should be considered integral components of AF management. Yet, the extent to which these psychosocial variables contribute to disease experience likely varies between patients, and it remains unclear which stressors are most relevant for whom.

Within this biopsychosocial framework, findings from the ARENA (Atrial Fibrillation Rhine-Neckar Region) study suggested that generalized anxiety predicts reduced HRQoL independent of medical comorbidities, underscoring the clinical importance of psychological variables in AF.^9^ However, it remains unclear whether and how psychosocial symptoms differentially affect patients across AF subgroups over time. A deeper understanding of these interrelations within patient-specific symptom networks could clarify commonalities and distinctions between AF subtypes, thereby supporting the development of more personalized treatment strategies within precision medicine.

Over the past decade, the application of network analyses in psychological and psychiatric research has increased substantially,^12–14^ offering a framework well suited to capture the complexity of symptom interactions in chronic medical conditions, such as diabetes,^15^ the SARS-COV2 disease,^16^ coronary heart disease, or heart surgery.^17^ Applying this approach to AF could reveal key mechanisms through which psychosocial processes influence HRQoL and disease burden, offering new targets for tailored interventions.^17,18^

Therefore, using data from the large, prospective ARENA cohort, we analysed psychosocial symptom networks across AF types and levels of symptom burden at baseline and 1-year follow-up to examine subgroup-specific constellations and their temporal stability. Specifically, we assessed whether network structure, connectivity, and node centrality differed between asymptomatic [European Heart Rhythm Association (EHRA = 1)] and symptomatic patients (EHRA ≥ 2), as well as across AF subtypes, and how these features evolved over time.

## Methods

### Study design and patient population

The ARENA project is a large, prospective, multicentre observational cohort initiated by the Foundation Institute for Cardiac Infarction Research (IHF) in Ludwigshafen, Germany, in cooperation with the cardiology departments of the hospitals in Ludwigshafen, Heidelberg, and Mannheim, affiliated outpatient cardiologists, and the Department of Clinical Pharmacology and Pharmacological Epidemiology at Heidelberg University Hospital.^19,20^ Adults aged 18 years or older with a confirmed diagnosis of AF, residence in the Rhine-Neckar region, and written informed consent were eligible for participation. Recruitment began in August 2016. Of more than 5000 individuals screened, 2777 patients were enrolled between August 2016 and December 2018.

For this secondary analysis, we included 996 participants who had complete baseline (T0) and 1-year follow-up (T1) data with an available EHRA symptom classification, and 924 participants with complete data for AF subtype comparisons (paroxysmal, persistent, or permanent). All procedures complied with the Declaration of Helsinki and followed the STROBE guidelines for observational research.^21^ The study was approved by the Ethics Committee of the Rhineland-Palatinate State Medical Association and the ethics boards of Heidelberg and Mannheim University Medical Centers. All participants provided written informed consent prior to participation. The primary study is registered at ClinicalTrials.gov under the identifier NCT02978248 (registered 30 November 2016).

### Measures

At baseline, demographic and clinical information was collected, including age, sex, AF subtype (paroxysmal, persistent, or permanent), history of cardioversion, history of catheter ablation, and comorbid conditions such as diabetes mellitus or structural heart disease, as described previously.^9,20^ Symptom burden related to AF was evaluated using the EHRA symptom classification, which quantifies the extent to which AF symptoms interfere with daily functioning.^22^ Psychosocial and quality-of-life variables were assessed both at baseline (T0) and at 1-year follow-up (T1). Generalized anxiety was measured using the Generalized Anxiety Disorder-2 (GAD-2) scale.^23^ This two-item questionnaire assesses how often during the past 2 weeks participants were bothered by feeling nervous, anxious, or on edge, and by being unable to stop or control worrying. Each item is rated on a four-point scale (0 = not at all, 1 = at several days, 2 = at more than half the days, 3 = at almost every day), with higher scores indicating greater generalized anxiety.

Cardiac-specific anxiety was assessed using a two-item version of the Cardiac Anxiety Questionnaire (CAQ-2).^24^ The two items capture (i) heart-related worry (‘When I have chest discomfort or when my heart is beating fast, I worry that I may have a heart attack.’) and (ii) heart-related avoidance (‘I avoid activities that make my heart go faster.’). Each item is rated on a five-point scale ranging from 0 (never) to 4 (always), with higher scores reflecting stronger cardiac anxiety.

Health-related quality of life was measured using the validated German version of the EQ-5D-5L instrument,^24^ which assesses mobility, self-care, usual activities, pain/discomfort, and anxiety/depression on five levels (no problems, slight, moderate, severe, and extreme problems). Index scores were calculated using the German EQ-5D-5L value set (range = −0.661–1), with higher values representing better HRQoL. To facilitate interpretation in the network analyses, the HRQoL index was inverted linearly so that higher values indicated lower HRQoL.

Psychosocial stressors (loneliness, financial strain, stress at home, noise-related stress, and sleep problems) were rated on a four-point scale from 0 (‘not at all’) to 3 (‘very much’). Supplementary material online, *Table S1* provides an overview of all psychosocial constructs, including their definitions and corresponding questionnaire items. All measures were available at both assessment points and were used to estimate cross-sectional and longitudinal network models. Reliabilities for the GAD-2, CAQ-2, and EQ-5D instruments were examined in the ARENA cohort as reported previously.^9^

### Statistical analyses

Compared to traditional regression or factor-analytic approaches, network models enable the simultaneous examination of interrelations among many variables, allowing a nuanced understanding of complex interrelationships in numerous fields.^25,26^ Network analyses are a novel set of statistical tools used to identify patterns of statistical associations between variables of psychopathology.^27,28^ Hereby, each clinical variable, such as worry, represents a *node* that can be connected to other nodes by *edges*. In cross-sectional data designs, the Gaussian graphical model (GGM) is often used to generate an undirected network, whereby the edges represent conditional associations between variables, while conditioning on all other variables. These are also referred to as partial correlations or *edge weights*.^29^  *Centrality* indices then quantify the relative importance of a node in a given network, such as *Strength* which ‘sums the absolute edge weights of edges per node’.^27^ Oftentimes, *central* nodes are theorized to have the greatest influence on the development and maintenance of a disorder and should thus be the primary targets of therapeutic interventions.^13,28,30,31^

### Comparing different European Heart Rhythm Association classes and atrial fibrillation subtypes

To examine differences in patient characteristics and outcomes, we stratified participants by AF type (paroxysmal vs. persistent/permanent) and symptom burden using the EHRA classification. European Heart Rhythm Association classes were dichotomized into asymptomatic (EHRA = 1) and symptomatic patients (EHRA ≥ 2), reflecting the clinically meaningful distinction between absence and presence of symptom-related impairment. This approach also ensured adequate sample size and statistical stability for network estimation.

This stratification allowed us to investigate differences in clinical parameters and treatment responses based on symptom burden and AF progression. It also supports the identification of patient subtypes that may benefit from more individualized treatment strategies in the context of precision medicine. Subgroups were analysed at two time points—baseline (T0) and 1-year follow-up (T1)—to evaluate patterns of symptom progression within and between groups.

### Network estimation

Statistical analyses were performed using R (version 4.3.3, R Core Team, 2024) or Jamovi (version 2.6.45). First, descriptive statistics were computed, and Wilcoxon rank-sum tests were used to examine group differences across EHRA classes and AF subtypes. We used the package bootnet (*estimateNetwork* function) to estimate the network models.^29^ Network models consist of nodes (variables) and edges (statistical relationships between nodes). In our case, an edge represents a (partial) correlation after conditioning on all other edges in the network, also called weight (*w*). We used Spearman rank-order correlations to account for the ordinal data.^32^ Also, we used the Gaussian Markov random field estimation using graphical *least absolute shrinkage and selection operator* (LASSO;^33^) and the extended Bayesian information criterion to select optimal regularization parameters with the hyperparameter set to default (γ = 0.5). No redundant nodes were identified using the function *goldbricker* with default parameter settings from the *networktools* package.^34^ We used *qgraph* to plot the networks,^35^ which uses the Fruchterman-Rheingold algorithm^36^ to place nodes with stronger associations closer to each other. To facilitate interpretation, the node placement layout of the first reference network (T0, lower class) was also used for plotting all other networks. We used the *radarchart* function of the *fmsb* package^37^ to draw radar charts. With those radar charts, we depicted the differences in Strength estimates by time point and EHRA class/AF subtype.

### Network inference

Importance of single nodes was formally assessed using the Strength centrality metric. Strength is the sum of absolute edge weights of one node with all its connected nodes. To compare groups [EHRA = 1 (asymptomatic) vs. EHRA ≥ 2 (symptomatic), AF = paroxysmal vs. AF = persistent or permanent] and time point (baseline T0 vs. 1-year follow-up T1), we created different networks for each individual combination and then performed a non-parametric network comparison test (NCT) with 1000 permutations. For this purpose, we used the package *NetworkComparisonTest* with the function NCT.^38^ Due to the exploratory nature of this paper, we did not adjust the alpha level.

### Network accuracy and stability

We tested the stability of edge weights by creating 2000 samples with the *bootnet* package^29^ using non-parametric bootstrapping (resampling rows with replacement). Its results can be found in the Supplementary material online, *Figures S1* and *S2*. Case-drop bootstrapping (leaving a predefined proportion of the sample out) with 2000 samples was then used to test how much per cent of our original sample could be dropped to maintain an average correlation of *r* > 0.7 of the node *Strength* centrality estimates between the original and bootstrapped samples—the correlational stability (CS)-coefficient (minimum ≥ 0.25, recommended: ≥0.5^29^). CS-coefficients are reported in the Results and respective figures can be found in the Supplementary material online, *Appendix* (Supplementary material online, *Figures S3*–*S10*).

## Results

### Sample characteristics

The dataset for the EHRA group consisted of 996 patients (mean age = 70.62, SD = 10.60, 673 male, 320 female), whereas the dataset of the AF group consisted of 924 (mean age = 70.45, SD = 10.57, 617 male, 304 female) patients. For a comprehensive overview of the sample characteristics refer to *Table 1* (*Table 1* shows differences for EHRA class (1 vs. ≥2), while Supplementary material online, *Table S3* shows differences for AF type (paroxysmal vs. persistent or permanent).

**Table 1:** Sample baseline characteristics of the study population, overall and separated by EHRA class

			EHRA class (MD = 1781)						
	EHRA sample total (*n* = 996)		1 (*n* = 592)		≥2 (*n* = 404)				
Categorical variables	*n*	%	*n*	%	*n*	%	*χ* ^2^	df	*P*
Sex (female) (MD = 3)	320	32.2	233	23.5	87	8.8	35.14	1	<0.001
Single (MD = 14)	196	20.0	130	13.2	66	6.7	2.47	1	0.116
University entrance qualification (MD = 12)	551	56.0	323	32.8	259	26.3	0.143	1	0.705
Organic heart diagnoses (MD = 22)	716	73.5	428	43.9	288	29.6	0.21	1	0.646
DM (MD = 9)	206	20.9	119	12.1	87	8.8	0.35	1	0.552
AF type (MD = 95)							12.98	2	0.002
Paroxysmal	565	62.7	352	39.1	213	23.6			
Persistent	223	24.8	149	16.5	74	8.2			
Permanent	113	12.5	53	5.9	60	6.7			
Chronic kidney disease (MD = 9)	141	14.3	91	9.2	50	5.1	1.68	1	0.194
Stroke/TIA (MD = 9)	116	11.8	65	6.6	51	5.2	0.68	1	0.408
New-onset AF (MD = 25)	102	10.5	72	7.4	30	3.1	5.19	1	0.023
Cardiovascular comorbidities									
Congestive heart failure (MD = 25)	176	18.1	65	6.7	111	11.4	1.18	1	0.276
Coronary artery disease (MD = 25)	320	33.0	131	13.5	189	19.5	0.03	1	0.872
Previous MI (MD = 25)	130	13.4	65	6.7	65	6.7	5.53	1	0.019
Hypertensive cardiomyopathy (MD = 25)	182	18.7	60	6.2	122	12.6	5.38	1	0.020
Peripheral artery disease (MD = 12)	51	5.2	21	2.1	30	3.0	0.02	1	0.901
AF treatment									
VKA (MD = 44)	189	19.9	88	9.2	101	10.6	4.34	1	0.037
DOAC (MD = 44)	647	68.0	235	24.7	412	43.3	10.88	1	<0.001
Beta-blocker (MD = 105)	666	74.7	260	29.2	406	45.6	0.24	1	0.624
Calcium channel blocker (MD = 105)	53	5.9	22	2.5	31	3.5	0.09	1	0.758
Cardiac glycoside (MD = 105)	82	9.2	33	3.7	49	5.5	0.02	1	0.886
Class I antiarrhythmic drug (MD = 143)	37	4.3	5	0.6	32	3.8	10.67	1	0.001
Class III antiarrhythmic drug (MD = 143)	53	6.2	19	1.9	34	4.0	0.26	1	0.611
Cardioversion (MD = 29)	160	16.5	53	5.5	107	11.1	4.86	1	0.028
CA (MD = 25)	141	14.6	29	3.0	112	11.6	28.36	1	<0.001
PM implantation (MD = 25)	35	3.6	15	1.6	20	2.1	0.05	1	0.815
ICD implantation (MD = 25)	16	1.7	6	0.6	10	1.0	0.08	1	0.777
History of cardioversion (MD = 45)	286	30.1	191	20.1	95	10.0	9.22	1	0.002
History of CA (MD = 45)	186	19.6	130	13.7	56	5.9	10.33	1	0.001

Continuous variables	*M*	SD	*M*	SD	*M*	SD	*t*	df	*P*
Age in years (MD = 13)	70.62	10.60	69.22	11.06	72.68	9.54	−5.09^a^	981	<0.001
EF (MD = 483)	53.72	12.65	54.79	12.06	51.71	13.50	2.64^a^	511	0.009
BMI (MD = 12)	28.78	12.21	29.15	11.49	27.42	13.20	1.16	982	0.247
CHA2DS2-VASC (MD = 42)	3.14	1.66	3.09	1.69	3.20	1.62	−0.98	952	0.327
HAS-BLED Score (MD = 57)	1.93	1.05	1.88	1.07	1.99	1.00	−1.60^a^	937	0.111
GAD-2 (MD = 0)	1.35	1.31	1.55	1.35	1.07	1.20	5.71^a^	994	<0.001
CAQ-2 (MD = 0)	2.95	1.83	3.38	1.72	2.32	1.80	9.33	994	<0.001
Stress at work (MD = 693)	1.59	0.69	1.61	0.70	1.55	0.67	0.60	301	0.547
EQ-5D-5L Index score (MD = 0)	0.83	0.23	0.81	0.23	0.85	0.22	−2.68	994	0.008
Duration of AF in years (MD = 223)	7.44	8.34	8.34	8.80	6.88	7.99	2.36	771	0.018

Differences by EHRA class in categorical variables were tested with *χ*^2^ tests of associations. Differences by EHRA class in continuous variables were tested with independent sample *t*-tests.

MD, missing data; AF, atrial fibrillation; BMI, body mass index; CA, catheter ablation; CAQ-2, Cardiac Anxiety Questionnaire 2-item screener; DM, diabetes mellitus; DOAC, direct oral anticoagulant; EF, ejection fraction; EQ5D-5L, EuroQoL-5D; GAD-2, Generalized Anxiety Disorder 2-item screener; ICD, implantable cardioverter defibrillator; MI, myocardial infarction; PM, pacemaker; TIA, transient ischaemic attack; VKA, vitamin K antagonist.

^a^Significant (*P* < 0.05) Levene’s test, suggesting violation of the assumption of equal variances.

### European Heart Rhythm Association class

#### Descriptive results and differences between subgroups and time points

At T0, EHRA classes differed with respect to all variables, besides stress at home and financial stress, such that patients with higher EHRA scores (≥2) reported higher burden. At T1, however, only heart-related avoidance (*P* = 0.014), heart-related worry (*P* < 0.001), loneliness (*P* = 0.001), and sleep problems (*P* = 0.027) differed significantly, such that patients with higher EHRA class (≥2) reported higher psychosocial burden. Time points did not differ in patients with lower EHRA class, but heart-related avoidance (*P* = 0.014) and heart-related worry (*P* < 0.001) decreased over time in patients with higher EHRA scores (≥2) (see *Table 2*).

**Table 2:** Descriptive results and Wilcoxon rank-sum tests split by time point and EHRA class

	T0 (baseline)						T1 (1-year follow-up)						EHRA = 1		EHRA ≥ 2	
Variable	EHRA = 1 (*n* = 404)		EHRA ≥ 2 (*n* = 592)		EHRA = 1 vs. EHRA ≥ 2		EHRA = 1 (*n* = 404)		EHRA ≥ 2 (*n* = 592)		EHRA = 1 vs. EHRA ≥ 2		T0 vs. T1		T0 vs. T1	
	Mean	SD	Mean	SD	*W*	*P*	Mean	SD	Mean	SD	*W*	*P*	*W*	*P*	*W*	*P*
Anxiety	0.60	0.66	0.91	0.78	93 814	<0.001*	0.68	0.75	0.80	0.81	93 814	0.773	4690	0.907	15 854.5	0.065
Heart-related avoidance	1.10	1.13	1.54	1.12	92 509	<0.001*	1.10	1.15	1.36	1.12	92 509	0.004*	10 286.5	1	33 633	0.014*
Heart-related worry	1.22	1.00	1.84	1.00	79 005.5	<0.001*	1.16	1.00	1.57	1.01	79 005.5	<0.001*	10 634	1	35 112	<0.001*
Inverted health-related quality of life	−0.42	0.22	−0.38	0.23	99 179	<0.001*	−0.40	0.24	−0.38	0.24	107 610	0.272	16 977.5	0.352	50 745.5	1
Loneliness	0.32	0.57	0.52	0.74	103 308	0.001*	0.31	0.61	0.48	0.71	103 308	0.001*	2107	1	6556.5	1
Financial stress	0.29	0.52	0.31	0.53	117 175.5	1	0.23	0.46	0.28	0.52	117 175.5	1	1587	0.146	4919	1
Stress at home	0.94	0.50	1.05	0.57	109 131	0.106	0.92	0.56	0.99	0.62	109 131	1	2949	1	10 326	0.513
Stress from noise	0.34	0.56	0.54	0.65	100 164.5	<0.001*	0.36	0.57	0.45	0.64	100 164.5	1	2308	1	9099	0.035
Sleep problems	0.95	0.81	1.24	0.86	96 509	<0.001*	1.00	0.85	1.21	0.91	96 509	0.027*	4963.5	1	17 379.5	1
Worry	0.47	0.66	0.64	0.71	103 281	0.002*	0.51	0.72	0.59	0.74	103 281	1	4138	1	13 600.5	1

T0, baseline; T1, 1-year follow-up; *W*, Wilcoxon test statistic; *P*, Bonferroni-adjusted *P-*value; EHRA, European Heart Rhythm Association Score.

^*^indicates significance at *P*  *<* 0.05.

#### Network descriptives

Overall network connectivity (number of present edges divided by number of possible edges) was rather low (lower EHRA class at T0 = 28.9%, lower EHRA class at T1 = 20.0%, higher EHRA class at T0 = 26.7%, higher EHRA class at T1 = 33.3%). This is not surprising, since LASSO regularization facilitates sparse network structures.

Correlation stability coefficients were at least acceptable (≥0.25) or larger than recommended (≥0.5^29^) lower EHRA class at T0 = 0.36, lower EHRA class at T1 = 0.59, higher EHRA class at T0 = 0.59, and higher EHRA class at T1 = 0.59. More information can be found in the Supplementary material online, *Appendix* (see Supplementary material online, *Figures S1*–*S4*).

The strongest edge according to weight in all networks was the one between *anxiety* and *worry* (*w* = 0.42–0.47). The second strongest edge for three networks (lower EHRA class at T0, lower EHRA class at T1, higher EHRA class at T1) was the one between *heart-related worry* and *heart-related avoidance* (*w* = 0.23–0.42). However, for the higher EHRA class at T0, it was the edge between *anxiety* and *heart-related worry* (*w* = 0.23) (see *Figure 1*).

**Figure 1:**
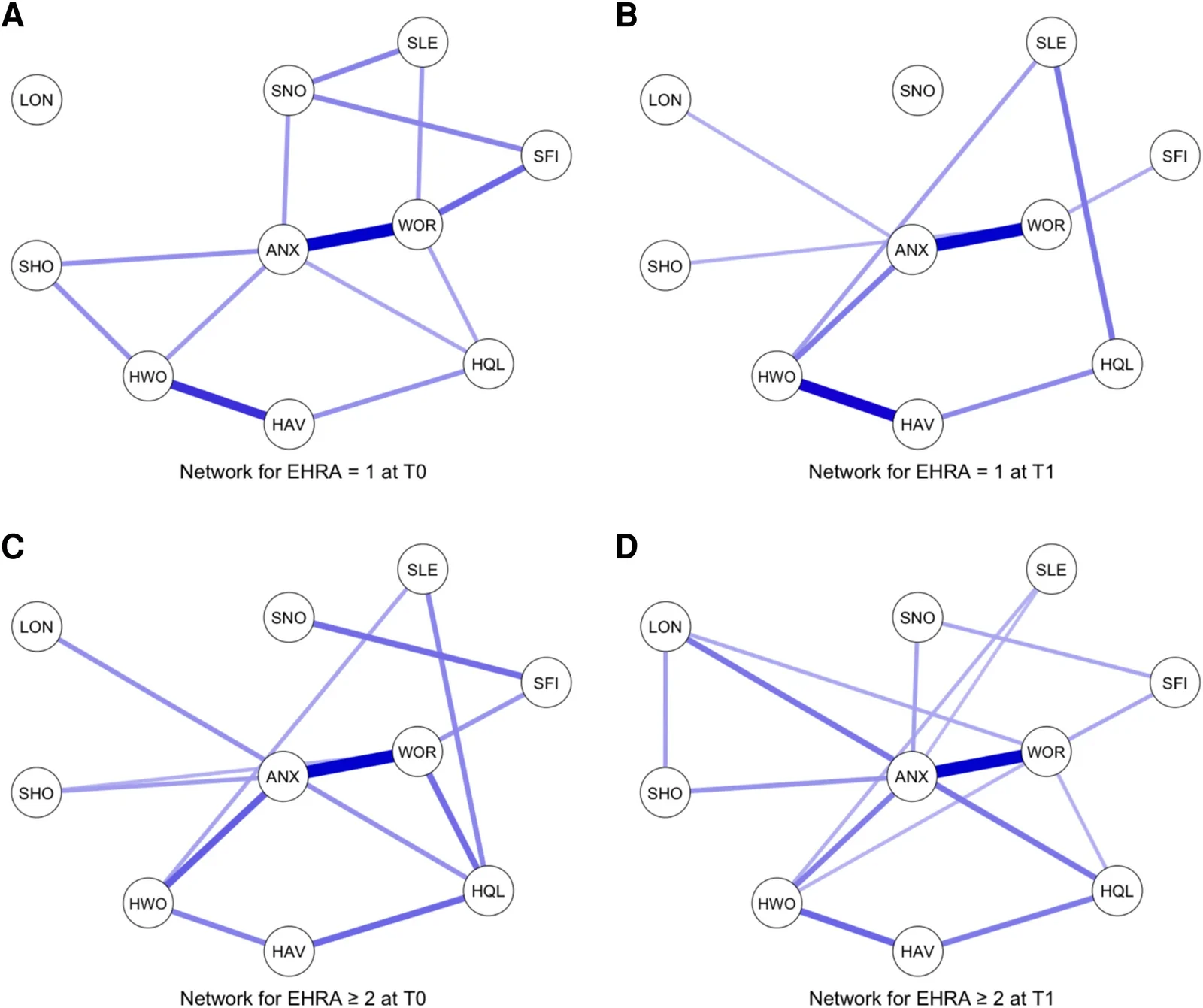
Network structure for EHRA classes by time point. (*A*) Network for EHRA class 1 at T0. (*B*) Network for EHRA class 1 at T1. (*C*) Network for EHRA class ≥ 2 at T0. (*D*) Network for EHRA class ≥ 2 at T1. All lines indicate positive edges. Thicker and more saturated edges indicate stronger connections. Nodes with stronger and/or more connections are placed closer to each other. Node placement of all networks depends on the layout of (*A*). EHRA, European Heart Rhythm Association; T0, baseline; T1, 1-year follow-up; ANX, anxiety; SHO, stress at home; SNO, stress from noise; SFI, financial stress; SLE, sleep disturbance; LON, loneliness; WOR, worry; HWO, heart-related worry; HAV, heart-related avoidance; HQL, inverted health-related quality of life index.

The most central nodes according to Strength in all four networks were *anxiety* (ANX), *worry* (WOR), *heart-related worry* (HWO), and *inverted health-related quality of life* (HQL): lower EHRA class at T0 = ANX (1.02), WOR (0.94), and HWO (0.62); lower EHRA class at T1 = ANX (0.80), HWO (0.78), and WOR (0.72); higher EHRA class at T0 = WOR (0.88), ANX (0.79), and HQL (0.73); and higher EHRA class at T1 = ANX (1.06), WOR (0.94), and HQL (0.66) (see *Figure 2* and Supplementary material online, *Figure S1*).

**Figure 2:**
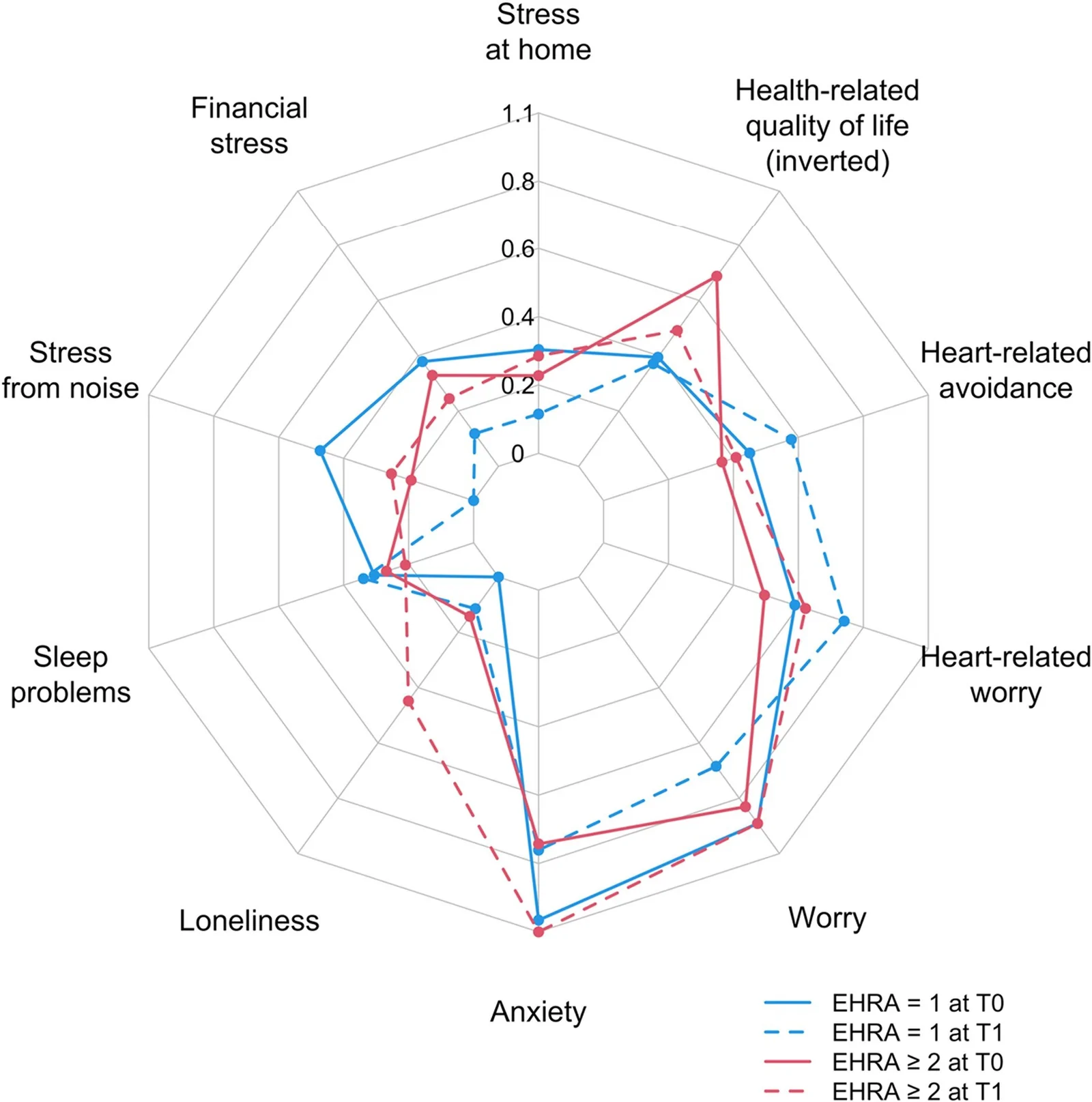
Radar chart: Strength centrality for EHRA classes by time point. EHRA, European Heart Rhythm Association score; T0, baseline; T1, 1-year follow-up. Strength is the sum of absolute edge weights of one node with all its connected nodes. There were no significant differences of the global Strength of nodes between EHRA classes at the same time point, nor between time points in the same EHRA class. Single node Strengths did not differ significantly between EHRA classes. However, for the high EHRA class, the node Strength of *loneliness* increased significantly (*P* = 0.041) from T0 (*S* = 0.151) to T1 (*S* = 0.474).

#### Differences between European Heart Rhythm Association classes at the same time point

The NCT indicated that the overall connectivity profiles of the networks were globally invariant between EHRA classes at the same time point (at T0: maximum difference in edge weights (*M*) = 0.181, *P* = 0.772, at T1: *M* = 0.211, *P* = 0.260). However, the following edges were not invariant: first, at T0, the edge between *stress at home* and *heart-related worry* was present for the lower EHRA class (*w* = 0.162), but absent for the higher EHRA class (*w* = 0, *P* < 0.001). Also, at T0, the edge between *heart-related worry* and *heart-related avoidance* at T0 was stronger for the lower EHRA class (*w* = 0.316) than for the higher EHRA class (*w* = 0.174, *P* = 0.025). And lastly, at T0, the edge between *sleep problems* and *worry* at T0 was present for the lower EHRA class (*w* = 0.142), but absent for the higher EHRA class (*w* = *0*, *P* = 0.027). Second, at T1, the edge between *heart-related avoidance* and *heart-related worry* at T1 was stronger for the lower EHRA class (*w* = 0.430) than for the higher EHRA class (*w* = 0.233, *P* = 0.003). Also, at T1, the edge between *sleep problems* and the *inverted health-related quality of life* was present for the lowest EHRA class (*w* = 0.211), but absent for the higher EHRA class (*w* = 0, *P* = 0.014) (see *Figure 1*).

Also, there was no significant difference of the global strength of nodes between EHRA classes at the same time point (at T0: global difference of Strength (*ΔS*) = 0.249, *P* = 0.667, at T1: *ΔS* = 0.545, *P* = 0.322). Likewise, single node Strengths did not differ significantly between EHRA classes (see *Figure 2* and Supplementary material online, *Figure S1*).

#### Differences between time points in the same European Heart Rhythm Association class

The NCT indicated that the overall structures of the networks were globally invariant between time points in the same EHRA class (EHRA = 1: *M* = 0.211, *P* = 0.437, EHRA ≥ 2: *M* = 0.162, *P* = 0.655). However, for the lower EHRA class, the following single edges were not invariant: first, the edge between *loneliness* and *anxiety* was absent at T0 (*w* = 0), but present at T1 (*w* = 0.121, *P* = 0.010). Second, the edge between *sleep problems* and the *inverted health-related quality of life* was absent at T0 (*w* = 0), but present at T1 (*w* = 0.211, *P* = 0.020). And third, the edge between *heart-related worry* and *heart-related avoidance* was lower at T0 (*w* = 0.316) than at T1 (*w* = 0.430, *P* = 0.050) (see *Figure 1*).

There was no significant difference of the global Strengths values of EHRA classes between time points in the same EHRA class (EHRA = 1: *ΔS* = 0.491, *P* = 0.318, EHRA ≥ 2: *ΔS* = 0.303, *P* = 0.319). Locally, however, for the high EHRA class, the Strength of *loneliness* increased significantly (*P* = 0.041) from T0 (*S* = 0.151) to T1 (*S* = 0.474) (see *Figure 2* and Supplementary material online, *Figure S1*).

### Atrial fibrillation type

#### Descriptive results and Wilcoxon rank-sum tests

At T0, AF types did not differ with respect to any variable. At T1, only heart-related worry differed significantly: patients with paroxysmal AF (*M* = 1.51) showed significantly higher worry than patients with persistent or permanent AF (*M* = 1.28, *P* = 0.041). Heart-related worry decreased significantly over time for both paroxysmal AF (*ΔM* = −0.16, *P* = 0.001), and persistent or permanent AF (*ΔM* = −0.23, *P* = 0.001) (see *Table 3*).

**Table 3:** Descriptive results and Wilcoxon rank-sum tests split by time point and atrial fibrillation type

	T0 (baseline)							T1 (1-year follow-up)							AF = paroxysmal (*n* = 582)			AF = persistent or permanent (*n* = 342)		
Variable	AF = paroxysmal (*n* = 582)		AF = persistent or permanent (*n* = 342)		AF = paroxysmal vs. AF = persistent or permanent			AF = paroxysmal (*n* = 582)		AF = persistent or permanent (*n* = 342)		AF = paroxysmal vs. AF = persistent or permanent			T0 vs. T1			T0 vs. T1		
	Mean	SD	Mean	SD	*W*	*P*		Mean	SD	Mean	SD	*W*	*P*		*W*	*P*		*W*	*P*	
Anxiety	0.85	0.77	0.73	0.72	107 221.5	1		0.80	0.82	0.68	0.75	107 538.5	0.953		13 830	1		4711	1	
Heart-related avoidance	1.35	1.13	1.50	1.17	92 280	1		1.26	1.16	1.28	1.12	97 978.5	1		25 658	1		11 837.5	0.063	
Heart-related worry	1.67	1.04	1.51	1.06	108 205.5	0.821		1.51	1.03	1.28	1.03	111 853	0.041	*	29 853	0.001	*	10 217.5	0.001	*
Inverted health-related quality of life	−0.40	0.22	−0.38	0.23	94 943	1		−0.39	0.23	−0.37	0.25	93 318	1		45 763.5	1		15 549.5	1	
Loneliness	0.46	0.71	0.42	0.67	101 246.5	1		0.45	0.70	0.36	0.66	106 803.5	0.945		4583	1		2574.5	1	
Financial stress	0.32	0.53	0.26	0.50	104 432	1		0.27	0.52	0.22	0.46	103 415.5	1		3942.5	0.747		1438	1	
Stress at home	1.03	0.56	0.95	0.54	105 401	1		1	0.62	0.91	0.58	105 182.5	1		7637	1		3618	1	
Stress from noise	0.50	0.64	0.37	0.57	109 348.5	0.137		0.44	0.63	0.35	0.57	105 671	1		7365	0.358		2177.5	1	
Sleep problems	1.10	0.85	1.15	0.88	97 071	1		1.13	0.89	1.10	0.90	101 437	1		13 277	1		5420	1	
Worry	0.62	0.72	0.52	0.66	106 408	1		0.60	0.77	0.49	0.69	106 657.5	1		12 135.5	1		3699	1	

T0, baseline; T1, 1-year follow-up; *W*, Wilcoxon test statistic; *P*, Bonferroni-adjusted *P-*value; AF, atrial fibrillation.

^*^indicates significance at *P*  *<* 0.05.

#### Network descriptives

Overall network connectivity (number of present edges divided by number of possible edges) was medium and showed larger variability compared to EHRA networks (paroxysmal AF at T0 = 33.3%, paroxysmal AF at T1 = 40.0%, persistent or permanent AF at T0 = 15.6%, persistent or permanent AF at T1 = 20.0%).

Correlation stability coefficients of Strength centrality estimates were at least acceptable (≥0.25) or larger than recommended (≥0.5^29^): paroxysmal AF at T0 = 0.36, paroxysmal AF at T1 = 0.52, persistent or permanent AF at T0 = 0.44, and persistent or permanent AF at T1 = 0.36. More information can be found in the Supplementary material online, *Appendix* (see Supplementary material online, *Figures S7*–*S10*).

Similar to the EHRA networks, the strongest edge according to weight in all networks was the one between *anxiety* and *worry* (*w* = 0.36–0.48). Also, the second strongest edge for all networks was the one between *heart-related avoidance* and *heart-related worry* (*w* = 0.22–0.31). The third strongest edge for three networks (paroxysmal AF at T0, paroxysmal AF at T1, persistent or permanent AF at T0) was the one between *heart-related worry* and *anxiety* (*w* = 0.21–0.24). However, for persistent or permanent AF at T1, it was the edge between *inverted health-related quality of life* and *heart-related avoidance* (*w* = 0.20) (see *Figure 3*).

**Figure 3:**
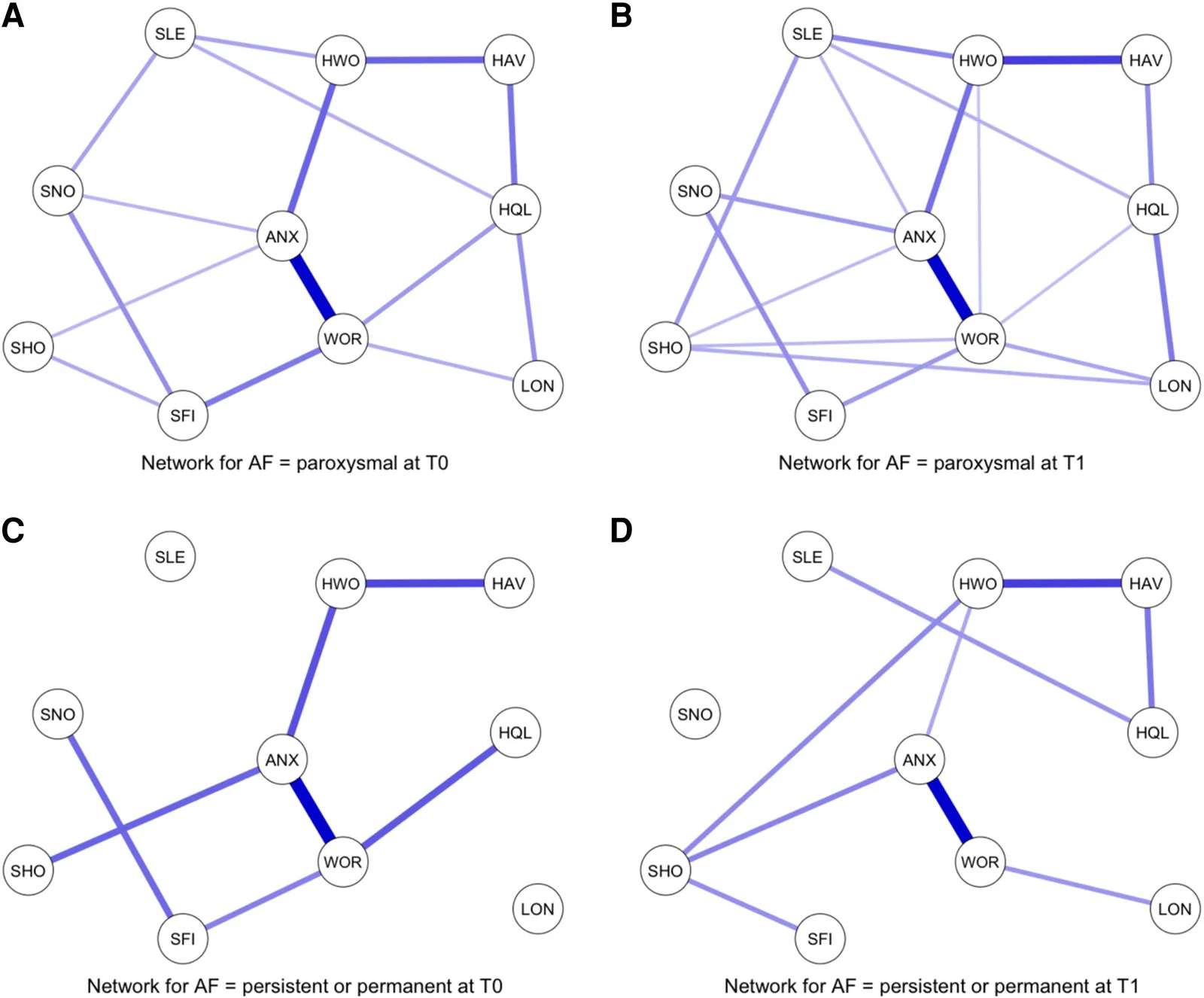
Network structure for atrial fibrillation types by time point. (*A*) Network for paroxysmal atrial fibrillation at T0. (*B*) Network for paroxysmal atrial fibrillation at T1. (*C*) Network for persistent or permanent atrial fibrillation at T0. (*D*) Network for persistent or permanent atrial fibrillation at T1. All lines indicate positive edges. Thicker and more saturated edges indicate stronger connections. Nodes with stronger and/or more connections are placed closer to each other. Node placement of all networks depends on the layout of (*A*). T0, baseline; T1, 1-year follow-up; ANX, anxiety; SHO, stress at home; SNO, stress from noise; SFI, financial stress; SLE, sleep disturbance; LON, loneliness; WOR, worry; HWO, heart-related worry; HAV, heart-related avoidance; HQL, inverted health-related quality of life index.

The most central nodes according to Strength in all four networks were *anxiety* (ANX), *worry* (WOR), *heart-related worry* (HWO), and *inverted health-related quality of life* (HQL): paroxysmal AF at T0 = ANX (0.97), WOR (0.95), and HQL (0.66); paroxysmal AF at T1 = ANX (0.75), WOR (0.70), and HWO (0.43); persistent or permanent AF at T0 = ANX (1.06), WOR (1.06), and HWO (0.80); and persistent or permanent AF at T1 = ANX (0.75), WOR (0.60), and HWO (0.59) (see *Figure 4* and Supplementary material online, *Figure S2*).

**Figure 4:**
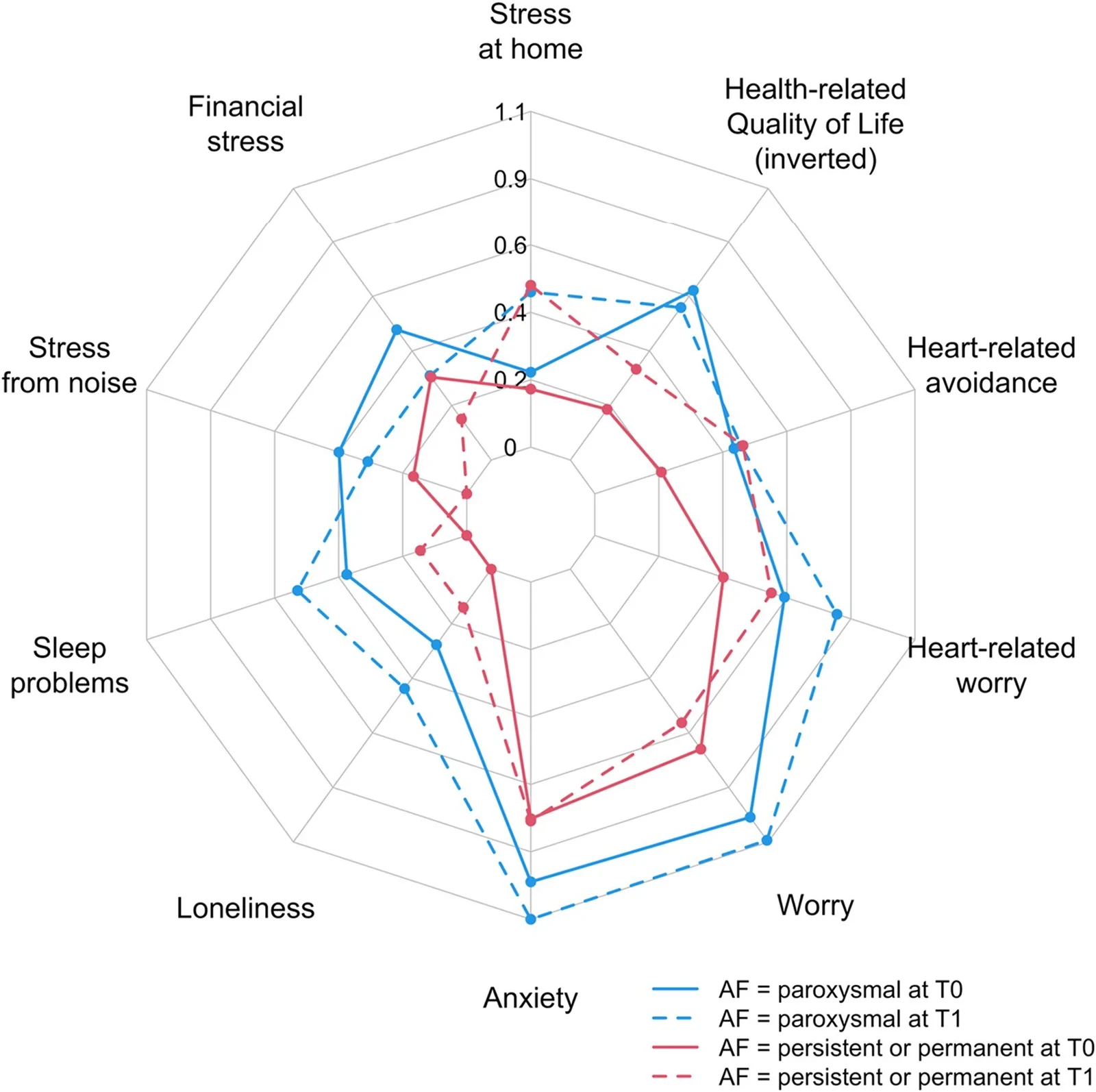
Radar chart: Strength centrality for atrial fibrillation types by time point. AF, atrial fibrillation; T0, baseline; T1, 1-year follow-up. Strength is the sum of absolute edge weights of one node with all its connected nodes. Significant differences of overall Strength between AF types at the same time point were found, such that persistent or permanent AF types generally showed weaker node Strengths (at T0: *ΔS* = 1.275, *P* = 0.034, at T1: *ΔS* = 1.21, *P* = 0.036). However, these global differences between AF types were not driven by significant differences in single node Strength values. There were no significant differences of the global Strength between time points with the same atrial fibrillation type and for single node Strength values.

#### Differences between atrial fibrillation types at the same time point

The NCT indicated that the overall structures of the networks were globally invariant between AF types at the same time point (at T0: *M* = 0.215, *P* = 0.428, at T1: *M* = 0.206, *P* = 0.535). However, the following single edges were not invariant: at T0, the edge between *anxiety* and *worry* was stronger for the paroxysmal AF type (*w* = 0.479) than for the persistent or permanent AF type (*w* = 0.357, *P* = 0.044). At T1, the edge between *stress at home* and *heart-related worry* was not present for the paroxysmal AF type (*w* = 0) but was present for persistent or permanent AF type (*w* = 0.170, *P* = 0.005) (see *Figure 3*).

Significant differences of overall Strength between AF types at the same time point were found, such that persistent or permanent AF types generally showed weaker node Strengths (at T0: *ΔS* = 1.275, *P* = 0.034, at T1: *ΔS* = 1.21, *P* = 0.036). These overall significant differences between AF types were not driven by differences in single node Strength values (*Table 4*) (see *Figure 4* and Supplementary material online, *Figure S2*).

**Table 4:** Node centrality differences (strength): paroxysmal vs. persistent or permanent atrial fibrillation

Variable	T0 (baseline)		T1 (1-year follow-up)	
	Difference in Strength	*P*	Difference in Strength	*P*
Anxiety	0.20	0.351	0.31	0.142
Heart-related avoidance	0.24	0.118	−0.01	0.942
Heart-related worry	0.46	0.104	0.24	0.361
Inverted health-related quality of life	0.20	0.266	0.22	0.201
Loneliness	0.29	0.275	0.32	0.149
Financial stress	0.18	0.376	0.17	0.521
Stress at home	0.05	0.798	−0.02	0.910
Stress from noise	0.40	0.155	0.41	0.096
Sleep problems	0.25	0.361	0.33	0.136
Worry	0.27	0.185	0.46	0.055

Strength is the sum of absolute edge weights of one node with all its connected nodes.

#### Differences between time points within the same atrial fibrillation type

The NCT indicated that the overall structures of the networks were globally invariant between time points within the same AF type (AF = paroxysmal: *M* = 0.156, *P* = 0.811, AF = persistent or permanent: *M* = 0.200, *P* = 0.572). Also, there were no significant differences of the global Strength between time points with the same AF type (AF = paroxysmal: *ΔS* = 0.326, *P* = 0.277, AF = persistent or permanent: *ΔS* = 0.391, *P* = 0.374). This was also the case for single node Strength values (see *Table 5*).

**Table 5:** Node centrality differences (Strength): T0 (baseline) vs. T1 (1-year follow-up)

Variable	Paroxysmal atrial fibrillation		Persistent or permanent atrial fibrillation	
	Difference in Strength	*P*	Difference in Strength	*P*
Anxiety	−0.12	0.508	−0.01	0.961
Heart-related avoidance	−0.02	0.719	−0.27	0.051
Heart-related worry	0.07	0.683	−0.16	0.619
Inverted health-related quality of life	−0.17	0.095	−0.16	0.479
Loneliness	−0.17	0.292	−0.15	0.560
Financial stress	0.18	0.166	0.16	0.440
Stress at home	−0.25	0.180	−0.33	0.071
Stress from noise	−0.16	0.402	−0.15	0.605
Sleep problems	0.10	0.525	0.18	0.335
Worry	−0.09	0.560	0.10	0.706

Strength is the sum of absolute edge weights of one node with all its connected nodes.

## Discussion

In this large, prospective AF cohort, psychosocial networks were globally stable across AF subtypes and time, but key edges and the centrality (importance) of specific nodes shifted locally. Across all models, the strongest connections emerged between anxiety and worry (GAD-2), cardiac worry and avoidance (CAQ-2), and anxiety and cardiac worry. Anxiety, general worry, cardiac worry, and HRQoL were consistently the most central variables. These results corroborate prior ARENA evidence that generalized anxiety prospectively predicts impaired HRQoL in AF^9^ and align with broader observations linking symptom burden to worse quality of life and outcomes.^4,9^ Anxiety’s central role in AF networks highlights the need for targeted psychoeducation and supportive interventions that help patients interpret symptoms accurately and reduce catastrophizing.

The prominence of anxiety-related nodes likely reflects both psychometric overlap and the genuine interplay between anxious arousal, cardiac-focused worry, and avoidance behaviours that can reinforce symptom perception and functional impairment. Importantly, the psychopathological process linking arousal, worry, and avoidance is well-described for panic disorder^39^ and heart-related panic disorder.^40^ Although the global network structures were stable, certain symptoms shifted in importance over time. Among symptomatic patients (EHRA ≥ 2), loneliness became more influential over time and sleep disturbance maintained a stable link to HRQoL, suggesting that both variables represent clinically relevant correlates of impaired well-being in AF. This pattern supports current guideline recommendations emphasizing patient-reported outcomes and a biopsychosocial model of AF care,^7^ as well as recent ESC consensus statements highlighting the central role of mental health in cardiovascular disease and its bidirectional interaction with cardiac conditions.^5^ However, the mechanisms underlying sleep disturbance in AF are likely multifactorial and may include both psychosocial distress and physiological contributors such as sleep-disordered breathing.

From a clinical perspective, these findings point to several potentially relevant intervention domains. The robust coupling between cardiac worry and avoidance points to the potential benefit of cognitive-behavioural interventions focused on exposure and cognitive restructuring to reduce maladaptive heart-related fear. Brief, AF-adapted CBT programmes may help patients reinterpret bodily sensations, reduce catastrophic thinking, and promote graded physical activity—approaches that are effective for anxiety across medical conditions^7^ and have recently been shown to improve AF-specific quality of life and reduce healthcare utilization in patients with symptomatic AF.^41^ Generalized anxiety, and particularly worry—a key driver of HRQoL impairment^42^—may be further addressed through CBT targeting repetitive negative thinking^43^ or through collaborative-care models integrated into routine cardiology.^44^

The rising centrality of loneliness over time suggests another therapeutic opportunity. Loneliness has been identified as an independent risk factor for cardiovascular morbidity and mortality, yet it remains under-recognized in cardiology settings.^45^ Previous network analyses, however, already identified loneliness as a central node to depression symptomatology in older adults.^46^ Structured social-connection interventions—such as group cardiac rehabilitation, peer support, or social prescribing—may mitigate this risk and improve psychosocial well-being. Additionally, the recurring association between sleep problems and reduced HRQoL underscores the importance of cognitive-behavioural therapy for insomnia, which has strong evidence of efficacy and can be delivered digitally or in brief formats.^47^ Early intervention on sleep and social isolation may prevent these symptoms from becoming chronic.

Together, these observations are consistent with a stepped-care model: routine screening of anxiety, cardiac worry, loneliness, and sleep disturbance (e.g. by using short instruments such as the GAD-2 and the CAQ-2), followed by interventions tailored to individual need and AF type. Future longitudinal and interventional studies should examine whether psychosocial domains identified as highly interconnected within the present networks are associated with subsequent changes in HRQoL and symptom burden. Such an approach could help operationalize guideline recommendations for patient-centred, outcome-based AF management, as emphasized in the ESC guidelines for AF, including the AF-CARE pathway and its focus on symptom burden and quality of life,^8^ and aligns with recent ESC consensus statements advocating the systematic integration of mental health into cardiovascular care.^5^

### European Heart Rhythm Association class

Subgroup analyses by symptom severity further refine this picture. Patients with higher EHRA scores (≥2) showed greater psychosocial burden at both time points. The mean of heart-related avoidance, heart-related worry, loneliness, and sleep problems consistently differed between classes. The higher EHRA class generally displayed denser networks at follow-up, whereas the lower EHRA class showed denser networks at baseline. Although the global network structures did not differ significantly between EHRA classes or across time, individual edges varied: for the lower EHRA class, specific connections—such as those between stress at home and heart-related worry at baseline, or between sleep problems and HRQoL at follow-up—were only present or stronger in the less burdened group. This suggests that domestic stress and sleep disturbances are more salient for mental health in patients with lower self-reported symptom burden. This could imply that these symptom associations may become chronic and gradually impair HRQoL.

At follow-up, the relationship between loneliness and anxiety, as well as between sleep problems and HRQoL, became more pronounced in the lower EHRA group. The increasing association between heart-related worry and avoidance at follow-up may reflect a well-known vicious cycle in anxiety and psychosomatic disorders: fear of cardiac symptoms promotes avoidance, which in turn reinforces anxiety which constitutes the key psychopathological mechanism of panic disorder.^48,49^ Even among patients with relatively low symptom burden, this dynamic may hinder adaptive coping. Oftentimes, panic disorder has only been investigated as a risk factor for AF and other cardiac diseases,^50,51^ but rarely with the reverse causality.^52^ As such, how psychological symptoms of moderately burdened cardiac patients develop after cardiac events remains understudied. In contrast, the higher EHRA class group showed no changes in symptom structure over time, though the node strength of loneliness increased significantly—indicating that loneliness may progressively embed itself as a chronic feature within the psychosocial symptom networks of more burdened patients.

### Atrial fibrillation type

At baseline, psychosocial symptom levels were largely comparable across AF types. At follow-up, however, patients with paroxysmal AF reported significantly higher heart-related worry than those with persistent or permanent AF, possibly reflecting less psychological adjustment to symptom recurrences. Heart-related worry decreased significantly over time in both AF groups, suggesting a general habituation effect independent of AF severity.

Globally, the structure of the networks remained similar between AF types and time points, though some local differences emerged. Initially, patients with paroxysmal AF exhibited a stronger link between anxiety and general worry, whereas at follow-up, patients with persistent or permanent AF showed an emerging association between domestic stress and heart-related worry. The overall psychosocial network connectivity was higher in paroxysmal AF, which may reflect a less stable pattern of psychosocial symptom interrelations early in the disease course. This pattern highlights a potential critical window for preventive interventions early in the disease course—particularly while AF is still paroxysmal—when psychosocial processes may be more responsive to change. The observed relevance of sleep disturbance and loneliness in earlier-stage AF suggests that these domains may represent potentially modifiable supportive targets, whereas more chronic AF presentations may require broader approaches addressing persistent psychosocial distress and maladaptive symptom-related cognitions.

### Clinical implications

From a clinical perspective, the present findings suggest that emotional distress, cardiac-focused worry, loneliness, and sleep disturbance represent interconnected psychosocial dimensions that are consistently associated with impaired HRQoL in AF. While these domains are conceptually overlapping, their persistent co-occurrence across AF subgroups and time points highlights the broader relevance of psychosocial burden within AF care. In routine clinical practice, such assessments may help contextualize patient-reported symptom burden beyond arrhythmia characteristics alone and identify patients who may benefit from additional supportive or psychosomatic care alongside standard AF management, including AF-adapted cognitive-behavioural approaches for anxiety-related processes, as well as targeted management of sleep disturbance and social disconnection where clinically indicated. These findings support integrated, patient-centred management approaches that incorporate psychosocial screening and patient-reported outcomes alongside rhythm-control strategies. Future studies should investigate which psychosocial and behavioural interventions are most effective for specific psychosocial symptom profiles and AF subtypes and whether targeting highly interconnected symptom domains can improve long-term symptom burden and HRQoL trajectories.

### Strengths and limitations

To our knowledge, this is the first study to investigate the interplay of psychosocial and quality-of-life variables in AF patients using a network approach. The large sample size and longitudinal measurement design provide a robust basis for exploratory inference. However, the NCTs have limited statistical power to detect small differences, and while the design included two assessment waves, causal relationships cannot be inferred. Notably, a substantial proportion of patients in our sample were classified as asymptomatic (EHRA 1; 41%). While this may appear high, similar proportions have been reported in large observational AF registries. For example, 39.7% of patients in the EORP-AF Pilot General Registry were classified as asymptomatic (EHRA 1).^53^ More broadly, a recent systematic review and meta-analysis estimated an overall prevalence of asymptomatic AF of 27%, but highlighted substantial heterogeneity across cohorts, with asymptomatic patients commonly accounting for approximately 20–50% of AF populations.^54^ This likely reflects the observational design of the ARENA cohort, which captures a broad, real-world spectrum of AF patients across diverse clinical settings. Importantly, the EHRA classification reflects perceived symptom-related impairment rather than arrhythmia burden *per se*, such that patients with documented AF may report no or minimal symptoms. This distribution should be considered when interpreting subgroup comparisons and the generalizability of the findings.

The observed network structures were inherently constrained by the psychosocial variables and questionnaire measures available within the ARENA cohort. Consequently, the networks reflect only the specific symptom domains assessed in this dataset rather than the full spectrum of psychosocial and clinical burden in AF. Inclusion of additional clinically relevant symptoms and characteristics—such as depressive symptoms, chest pain, fatigue, dyspnoea, or other comorbid conditions—might have altered network topology and centrality estimates. In particular, depressive symptoms may represent an important additional domain given their high prevalence in cardiovascular populations and their established association with adverse cardiovascular outcomes.^55^ Although information on prior AF-specific interventions was available, additional stratification by intervention status was not feasible because it would have resulted in insufficient sample sizes for stable network estimation. Importantly, the assessed sleep problems may not solely reflect psychosocial distress. Sleep-disordered breathing, particularly obstructive sleep apnoea, is highly prevalent in AF populations and may contribute to impaired sleep quality and daytime symptoms.^56^ At the same time, symptom perception and AF-related disability are also shaped by psychosocial processes such as symptom hypervigilance and avoidance behaviour.^41^ Because detailed sleep phenotyping was not available in the ARENA cohort, the mechanisms underlying reported sleep disturbances could not be differentiated in the present analyses. Accordingly, the present findings should be interpreted as reflecting a specific subset of psychosocial symptom domains rather than a comprehensive representation of psychosocial burden in AF. Variable selection was further constrained by the available data and by the fact that the original ARENA study was not specifically designed for network analytic approaches. Furthermore, several psychosocial constructs were assessed using abbreviated two-item screening instruments (e.g. GAD-2 and CAQ-2), which may not fully capture the complexity and phenomenological nuance of generalized anxiety and cardiac-focused anxiety compared with longer measures.

The generalizability of our results is insofar limited as patients that were lost to follow-up and whose data did thus not contribute to the network analyses were significantly more likely to be female, older, single, highly educated, and burdened, although some mean differences were marginal (cf., Supplementary material online, *Table S2*). Upcoming studies should aim to ensure balanced samples and reach out more vigorously to these marginalized groups.

Generally, different network parameters do not necessarily equal differences in burden. As such, a network from a marginally burdened sample may also exhibit more and stronger connections than a network representing a highly burdened sample. This is why interpreting mean differences is also important. In many cases, traditional analyses of differences in means and network analytic techniques can complement each other. While we employed some statistical analyses of differences in means, future studies could expand on our work by combining network analysis with more sophisticated techniques.

The dichotomization of EHRA classes into asymptomatic vs. symptomatic patients may have obscured more nuanced differences across the full spectrum of AF symptom severity. Future studies should investigate psychosocial symptom networks across all EHRA categories to better capture potential non-linear or stage-specific effects e.g. by considering moderated network analyses^57^ in even larger samples. Furthermore, relying on clinical symptomatology (EHRA) or AF types (paroxysmal, persistent, permanent) alone does not suffice as a surrogate for AF burden, since various medical, psychological, or other conditions contribute to their reporting. This is why future studies should also include AF burden as a continuous variable, quantified by continuous electrocardiographic assessment.^58^

A further limitation of the present study is that AF type was assessed retrospectively based on available medical records and documented clinical diagnoses, which may have introduced some degree of misclassification.

Overall, the present analysis should be considered exploratory and hypothesis-generating rather than confirmatory. Replication in larger longitudinal cohorts, ideally including broader psychosocial and somatic phenotyping as well as panel or multilevel network approaches, will be important to determine the robustness and clinical utility of the observed network structures. Because inclusion in the analytic sample required complete longitudinal data, selection and attrition bias cannot be excluded. The analysed cohort may therefore over-represent patients with greater clinical stability, higher adherence, or lower psychosocial burden, potentially limiting the generalizability of the observed network structures to broader AF populations.

## Conclusion

In summary, anxiety, cardiac worry, avoidance, loneliness, and sleep disturbance form an interconnected psychosocial core in AF that remains globally stable but offers clear intervention entry points. Incorporating targeted psychological and behavioural modules into AF care—alongside guideline-recommended rhythm and rate management—could meaningfully improve patient-reported outcomes and quality of life.

## Supplementary Material

oeag166_Supplementary_Data

## Data Availability

The ARENA dataset generated and/or analysed for the present article is available upon request to the corresponding author.
