# Supplementary material for: Psychosocial symptom networks across atrial fibrillation subtypes: evidence from two waves of the ARENA cohort
Source: Eur Heart J Open. 2026 Oct 8;6(6):oeag166. doi: 10.1093/ehjopen/oeag166 (PMC13739053; doi:10.1093/ehjopen/oeag166)
Supplement: Supplementary file 1 [file oeag166_supplementary_data.docx]

# **Appendix**

Supplemental Figure 1: Strength Centrality for EHRA Classes by Time Point

*Note*. Strength is the sum of absolute edge weights of one node with all its connected nodes. Small numbers on the y-axis indicate the proportion of times parameters were estimated to be zero. Bold red lines indicate original sample values, black lines indicate mean of 2000 bootstrapped samples with 95% confidence interval indicated by shaded grey areas.

EHRA = European Heart Rhythm Association, T0 = baseline, T1 = 1-year follow-up, ANX = anxiety, SHO = stress at home, SNO = stress from noise, SFI = financial stress, SLE = sleep disturbance, LON = loneliness, WOR = worry, HWO = heart-related worry, HAV = heart-related avoidance, HQL = inverted health-related quality of life index

Supplemental Figure 2**:** *Strength Centrality for Atrial Fibrillation Types by Time Point*

*Note*. Strength is the sum of absolute edge weights of one node with all its connected nodes. Small numbers on the y-axis indicate the proportion of times parameters were estimated to be zero. Bold red lines indicate original sample values, black lines indicate mean of 2000 bootstrapped samples with 95% confidence interval indicated by shaded grey areas.

T0 = baseline, T1 = 1-year follow-up, ANX = anxiety, SHO = stress at home, SNO = stress from noise, SFI = financial stress, SLE = sleep disturbance, LON = loneliness, WOR = worry, HWO = heart-related worry, HAV = heart-related avoidance, HQL = inverted health-related quality of life index

Supplemental Figure 3: Strength case-dropping bootstrap stability in lower EHRA class at T0

Supplemental Figure 4: Strength case-dropping bootstrap stability in lower EHRA class at T1

Supplemental Figure 5: Strength case-dropping bootstrap stability in higher EHRA class at T0

Supplemental Figure 6: Strength case-dropping bootstrap stability in higher EHRA class at T1

Supplemental Figure 7: Strength case-dropping bootstrap stability in paroxysmal AF type at T0

Supplemental Figure 8: Strength case-dropping bootstrap stability in paroxysmal AF type at T1

Supplemental Figure 9: Strength case-dropping bootstrap stability in persistent of permanent AF type at T0

Supplemental Figure 10: Strength case-dropping bootstrap stability in persistent of permanent AF type at T1

**Table S1. Psychosocial constructs, definitions, and corresponding questionnaire items**

| **Construct (questionnaire)** | **Definition** | **Question wording** | **Response options** |
| --- | --- | --- | --- |
| Anxiety (GAD-2) | General feelings of nervousness, tension, or being on edge | Over the last 2 weeks, how often have you been bothered by the following problems? Feeling nervous, anxious or on edge | not at all (0) / on several days (1) / on more than half the days (2) / almost every day (3) |
| Worry  (GAD-2) | Repetitive, uncontrollable concerns about potential negative outcomes | Over the last 2 weeks, how often have you been bothered by the following problems? Not being able to stop or control worrying | not at all (0) / on several days (1) / on more than half the days (2) / almost every day (3) |
| Heart-related worry (CAQ) | Fear that cardiac symptoms indicate serious cardiac events | When I have chest discomfort or when my heart is beating fast, I worry that I may have a heart attack | never (0) / rarely (1) / sometimes (2) / often (3) / always (4) |
| Heart-related avoidance (CAQ) | Avoidance of activities that increase heart rate due to fear | I avoid activities that make my heart go faster | never (0) / rarely (1) / sometimes (2) / often (3) / always (4) |
| Loneliness | Subjective feeling of social isolation or lack of meaningful connection | How strongly do you experience loneliness? | not at all (0) / some (1) / quite (2) / very much (3) |
| Financial stress | Perceived strain related to financial difficulties | How strongly do you experience stress for financial reasons? | not at all or barely (0) / moderately (1) / very strongly or severely (2) |
| Stress at work | Perceived stress related to occupational demands | In the last four weeks, how often did you experience stress at work? | never (0) / sometimes (1) / often (2) / continuously (3) |
| Stress at home | Perceived stress from the domestic environment | In the last four weeks, how often did you experience stress at home? | never (0) / sometimes (1) / often (2) / continuously (3) |
| Stress from noise | Distress caused by environmental noise | How strongly do you experience stress because of noise? | not at all or barely (0) / moderately (1) / very strongly or severely (2) |
| Sleep problems | Difficulties initiating or maintaining sleep | In the last four weeks, how strongly did you feel impaired by problems with falling or staying asleep? | not at all (0) / some (1) / quite (2) / very much (3) |

**Table S2. Comparison between patients lost to follow-up with patients included in final analysis**

|  | **Total Sample (n = 2777)** | | **Dropout (n = 1781)** | | **Included in Analysis (n = 996)** | | **Test Statistics** | | | ***Interpretation*** |
| --- | --- | --- | --- | --- | --- | --- | --- | --- | --- | --- |
| **Categorical Variables** | ***n*** | ***%*** | ***n*** | ***%*** | ***n*** | ***%*** | ***χ2*** | ***df*** | ***p*** | ***Dropout is sig. more likely for*** |
| Sex (female) (MD = 31) | 1025 | 37.3 | 705 | 25.7 | 320 | 11.7 | 17.30 | 1 | < .001 | females |
| Single (MD = 1176) | 405 | 22.7 | 209 | 11.7 | 196 | 11.0 | 9.27 | 1 | .002 | singles |
| University entrance qualification (MD = 1013) | 930 | 52.7 | 379 | 21.5 | 551 | 31.2 | 9.57 | 1 | .002 | highly educated |
| EHRA class ≥ 2 (MD = 290) | 1081 | 43.5 | 677 | 27.2 | 404 | 16.2 | 5.70 | 1 | .017 | patients with higher EHRA class |
| AF type persistent or permanent (MD = 395) | 1443 | 60.6 | 878 | 36.9 | 565 | 23.7 | 2.75 | 1 | .097 |  |
| **Continuous Variables** | ***M*** | ***SD*** | ***M*** | ***SD*** | ***M*** | ***SD*** | ***t*** | ***df*** | ***p*** |  |
| Age (MD = 80) | 72.50 | 10.96 | 73.57 | 11.02 | 70.62 | 10.60 | 6.80 | 2695 | < .001 | older patients |
| BMI (MD = 925) | 28.43 | 10.12 | 28.03 | 7.03 | 28.78 | 12.21 | -1.60 | 1850 | .110 |  |
| EQ-5D-5L Index score (MD = 980) | 0.8 | 0.25 | 0.77 | 0.28 | 0.83 | 0.23 | -5.01^a^ | 1795 | < .001 | lower quality of life |

*Note.* MD = missing data; AF = atrial fibrillation; BMI = body mass index; EQ5D-5L = EuroQoL-5D; Differences by EHRA class in categorical variables were tested with χ2 tests of associations. Differences by EHRA class in continuous variables were tested with independent sample t-tests.

^a^ Significant (p < .05) Levene's test, suggesting violation of the assumption of equal variances.

**Table S3. Sample Baseline Characteristics of the Study Population, Overall and Separated by AF Type**

|  |  |  | **AF Type (MD = 1853)** | | | |  |  |  |
| --- | --- | --- | --- | --- | --- | --- | --- | --- | --- |
|  | **AF Type Sample Total (n = 924)** | | **paroxysmal (n = 342)** | | **persistent or permanent (n = 582)** | |  |  |  |
| **Categorical Variables** | ***n*** | ***%*** | ***n*** | ***%*** | ***n*** | ***%*** | ***χ2*** | ***df*** | ***p*** |
| Sex (female) (MD = 3) | 304 | 33.0 | 92 | 10.0 | 212 | 23.0 | 8.90 | 1 | .003 |
| Single (MD = 7) | 180 | 19.8 | 67 | 7.3 | 119 | 13.0 | 0.20 | 1 | .656 |
| University entrance qualification (MD = 11) | 511 | 56.0 | 191 | 20.9 | 320 | 35.0 | 0.11 | 1 | .742 |
| Organic heart diagnoses (MD = 20) | 675 | 74.7 | 272 | 30.1 | 403 | 44.6 | 11.17 | 1 | < .001 |
| DM (MD = 8) | 191 | 20.9 | 93 | 10.2 | 98 | 10.7 | 13.57 | 1 | < .001 |
| EHRA class ≥ 2 (MD = 23) | 347 | 38.5 | 134 | 14.9 | 213 | 23.6 | 0.42 | 1 | .515 |
| Chronic kidney disease (MD = 8) | 131 | 14.3 | 57 | 6.2 | 74 | 8.1 | 2.58 | 1 | .108 |
| Stroke/TIA (MD = 8) | 105 | 11.5 | 41 | 4.5 | 64 | 7.0 | 0.17 | 1 | .682 |
| New-onset AF (MD = 7) | 103 | 11.2 | 23 | 2.5 | 80 | 8.7 | 11.11 | 1 | < .001 |
| *Cardiovascular comorbidities* |  |  |  |  |  |  |  |  |  |
| Congestive heart failure (MD = 23) | 163 | 18.1 | 88 | 9.8 | 75 | 8.3 | 6.30 | 1 | .012 |
| Coronary artery disease (MD = 23) | 306 | 34.0 | 196 | 21.8 | 110 | 12.2 | 0.42 | 1 | .517 |
| Previous MI (MD = 23) | 126 | 14.0 | 89 | 9.9 | 37 | 4.1 | 4.04 | 1 | .044 |
| Hypertensive cardiomyopathy (MD = 23) | 169 | 18.8 | 85 | 9.4 | 84 | 9.3 | 13.44 | 1 | < .001 |
| Peripheral artery disease (MD = 11) | 51 | 5.6 | 34 | 3.7 | 17 | 1.9 | 0.39 | 1 | .531 |
| *AF treatment* |  |  |  |  |  |  |  | 1 |  |
| VKA (MD = 42) | 175 | 19.8 | 90 | 10.2 | 85 | 9.6 | 10.16 | 1 | .001 |
| DOAC (MD = 42) | 604 | 68.5 | 371 | 42.1 | 233 | 26.4 | 0.19 | 1 | .665 |
| Beta-blocker (MD = 82) | 636 | 75.5 | 383 | 45.5 | 253 | 30.0 | 1.24 | 1 | .266 |
| Calcium channel blocker (MD = 82) | 53 | 6.3 | 26 | 3.1 | 27 | 3.2 | 3.56 | 1 | .059 |
| Cardiac glycoside (MD = 82) | 81 | 9.6 | 26 | 3.1 | 55 | 6.5 | 32.17 | 1 | < .001 |
| Class I antiarrhythmic drug (MD = 117) | 37 | 4.6 | 30 | 3.7 | 7 | 0.9 | 6.67 | 1 | .010 |
| Class III antiarrhythmic drug (MD = 117) | 48 | 5.9 | 31 | 3.8 | 17 | 2.1 | 0.30 | 1 | .584 |
| Cardioversion (MD = 23) | 150 | 16.6 | 90 | 10.0 | 60 | 6.7 | 0.47 | 1 | .491 |
| CA (MD = 23) | 131 | 14.5 | 86 | 9.5 | 45 | 5.0 | 0.65 | 1 | .419 |
| PM implantation (MD = 23) | 31 | 3.4 | 20 | 2.2 | 11 | 1.2 | 0.06 | 1 | .812 |
| ICD implantation (MD = 23) | 13 | 1.4 | 7 | 0.8 | 6 | 0.7 | 0.42 | 1 | .517 |
| History of cardioversion (MD = 45) | 265 | 30.1 | 124 | 14.1 | 141 | 16.0 | 13.74 | 1 | < .001 |
| History of CA (MD = 45) | 168 | 19.1 | 66 | 7.5 | 102 | 11.6 | 0.30 | 1 | .586 |
| **Continuous Variables** | ***M*** | ***SD*** | ***M*** | ***SD*** | ***M*** | ***SD*** | ***t*** | ***df*** | ***p*** |
| Age (MD = 9) | 70.45 | 10.58 | 72.21 | 9.44 | 69.42 | 11.0 | 3.89^a^ | 913 | < .001 |
| EF (MD = 401) | 53.71 | 12.73 | 52.83 | 11.94 | 54.35 | 13.26 | -1.35 | 521 | .177 |
| BMI (MD = 12) | 28.88 | 12.56 | 29.97 | 19.24 | 28.24 | 5.66 | 2.01 | 910 | .045 |
| CHA2DS2-VASC (MD = 35) | 3.13 | 1.67 | 3.30 | 1.65 | 3.03 | 1.67 | 2.37 | 887 | .018 |
| HAS-BLED Score (MD = 49) | 1.90 | 1.03 | 2.01 | 1.04 | 1.83 | 1.03 | 2.44 | 873 | .015 |
| GAD-2 (MD = 0) | 1.39 | 1.32 | 1.26 | 1.24 | 1.47 | 1.36 | -2.32 | 922 | .021 |
| CAQ-2 (MD = 0) | 3.02 | 1.83 | 3.01 | 1.85 | 3.02 | 1.82 | -0.08 | 922 | .935 |
| Stress at work (MD = 625) | 1.58 | 0.68 | 1.45 | 0.69 | 1.65 | 0.67 | -2.48 | 297 | .014 |
| EQ-5D-5L Index score (MD = 0) | 0.82 | 0.23 | 0.81 | 0.23 | 0.83 | 0.22 | -1.36 | 922 | .175 |
| Duration of AF in years (MD = 196) | 7.25 | 7.98 | 7.14 | 7.98 | 7.40 | 7.98 | -0.42 | 726 | .672 |

*Note.* MD = missing data; AF = atrial fibrillation; BMI = body mass index; CA = catheter ablation; CAQ-2 = Cardiac Anxiety Questionnaire 2-item screener; DM = diabetes mellitus; DOAC = direct oral anticoagulant; EF = ejection fraction; EQ5D-5L = EuroQoL-5D; GAD-2 = Generalized Anxiety Disorder 2-item screener; ICD = implantable cardioverter defibrillator; MI = myocardial infarction; PM = pacemaker; TIA = transient ischemic attack; VKA = vitamin K antagonist. Differences by AF type in categorical variables were tested with χ2 tests of associations. Differences by AF type in continuous variables were tested with independent sample t-tests.

^a^ Significant (p < .05) Levene's test, suggesting violation of the assumption of equal variances.
